# Perceptions of nurses on human papillomavirus vaccinations in the Republic of Korea

**DOI:** 10.1371/journal.pone.0211475

**Published:** 2019-02-06

**Authors:** Hae Won Kim, Hyang Yuol Lee, Seong Eun Kim, Hye Young Ahn, Yeon Hee Kim, Young Jin Lee

**Affiliations:** 1 Research Institute of Nursing Science, Seoul National University College of Nursing, Seoul, Korea; 2 Seoul National University College of Nursing, Seoul, Korea; 3 Department of Nursing, Woosuk University, Jeollabuk-do, Korea; 4 College of Nursing, Eulji University, Daejeon, Korea; 5 Department of Nursing, Asan Medical Center, Seoul, Korea; University of Campania, ITALY

## Abstract

**Background:**

In June 2016, the Republic of Korea included free human papillomavirus (HPV) vaccinations for all 12-year-old girls in its national immunization program.

**Purpose:**

This study investigated perceptions of nurses on HPV vaccination and their intent to vaccinate preteens at the best ages.

**Methods:**

Recruited for the survey were 514 health teachers (181, 35.2%), public health nurses (168, 32.7%), and clinical nurses (165, 32.1%). Factor-analysis was conducted to validate the Vaccine-Hesitancy Scale for Korean nurses. Related variables associated with vaccine-acceptance were examined using the Kruskal–Wallis test and Spearman’s rho coefficients, due to lack of normalization.

**Results:**

Factor-analysis results showed that two factors of positive acceptance (7 items) and negative acceptance (3 items) accounted for 67.46% of the total variance, and explained 47.4% and 20.1%, respectively. Nurses who positively accepted HPV vaccine differed significantly in agreement to vaccinate girls or boys. For the proper vaccination age, a significant difference emerged between answers for girls and vaccine-acceptance scores, whereas no difference emerged between answers for boys and the scores. The vaccinated status of respondents significantly related to higher HPV vaccine acceptance, although age, religion, marital status, education, and working duration did not.

**Conclusions:**

This study showed that vaccine-acceptance levels reflect nurses’ attitudes and opinions about HPV vaccination for girls and boys.

## Introduction

The World Health Organization (WHO) raised vaccine-hesitancy—the hesitancy, delay, or refusal of vaccinations—as a global emerging concern, despite the availability of vaccination services [1–3]. Vaccine-hesitancy is present when vaccine-acceptance is lower than expected and is a country-specific phenomenon varying across time, place, and vaccines [3, 4]. Human papillomavirus (HPV) vaccine has been known as a cost-effective primary intervention to protect adolescents of both sexes from HPV infection and prevent several types of HPV-associated diseases such as genital warts and HPV-attributable (cervical/oropharyngeal/vulvar/vaginal/anal/penile) cancers [5–7]. However, a relatively lower uptake was found for the vaccine [8–10] among adolescent boys than girls in the United States [9, 11, 12]. HPV vaccination is most effective when given before one’s sexual debut (11–13 years), whereas vaccine uptake is clearly dependent on parental decisions for their children [12]. Vaccine safety was a major concern perceived by hesitant and antivaccine parents [13].

To identify factors on the effectiveness of vaccination programs, knowledge of the disease and the attitude of parents to vaccinate their children [14] are necessary. Parents who perceived benefit from the vaccine [12] were those deciding to provide HPV vaccinations to their children. For parents of sons, however, HPV vaccination was more likely when parents perceived higher risk for their sons’ HPV infection, having recommendations from health care providers, and parental perceptions about the importance of protecting their sons’ future partner [12]. More hesitant attitudes emerged among parents who were concerned about side effects and not sure about the safety and effectiveness of the vaccine [5, 12]; other barriers to the vaccination were cost and lack of healthcare for adolescents [12]. Willingness to accept HPV vaccine uptake could be affected by economic factors, a supportive culture of vaccination, and general beliefs of vaccine safety and efficacy [15].

Many countries adopted HPV vaccination in their national immunization programs for 11- or 12-year girls, and in some countries for children of both sexes [5]. Recommendation at the national level on educating health professionals and providing homogenous information about HPV vaccinations could be an effective response to the safety concerns of hesitant parents [13]. Information sources and confidence toward health professionals were the main determining factors of acceptance of the mandatory vaccination program in Italy [13]. Further, appropriate communication between parents and healthcare providers would facilitate reassurance for hesitant parents considering of HPV vaccination for their children [13]. Lack of knowledge about the vaccine among parents, coupled with an overestimation of parental vaccine hesitancy among providers, also hindered vaccine uptake [6, 16].

The HPV vaccination program has been globally implemented in 71 countries as a national immunization program [17]. As one of the countries, the Republic of Korea also started providing free HPV vaccinations in June 2016 for 12-year-old girls (born between January 1, 2003 and December 31, 2004) and one-to-one healthcare counseling services were offered to adolescent girls and their mothers who visited public health centers with any questions about improving women’s health [18, 19].

Healthcare-provider recommendations are a key means to offer adolescent immunizations for HPV [11, 20]. Providers’ recommendations highly influenced more than 70% of adolescents to receive HPV vaccination [11, 21, 22]. Similarly, receiving a provider recommendation was a stronger predictor of HPV vaccination than other factors, such as race/ethnicity, insurance coverage, knowledge about HPV, or perceptions regarding HPV vaccine effectiveness and safety [22, 23]. Similarly, a lack of provider recommendation was consistently found to be a major barrier to increasing the uptake of HPV vaccination [6]. In France, general practitioners diverged in immunization attitudes toward their relatives and patients, especially when considering the newest and most controversial vaccines; benefits of the HPV vaccine were the main focus of controversies [24, 25]. Even the majority of providers serving the highest risk populations for HPV infection and HPV-related cancers do not routinely recommend HPV vaccines to their patients [26]. Knowledge of primary care professionals affects the information provided to patients and is fundamental in the decision-making of vaccine uptake [10]. To promote HPV vaccine-acceptance, healthcare-providers caring for target populations should be confident about recommending HPV vaccinations and perceive themselves as opinion leader in providing health advice [16]. HPV vaccine uptake could be enhanced by the strong recommendation of the vaccine as a safe, routine immunization that prevents cancer, coadministered with other vaccines [6]. In some cases, scheduling a reminder and recall results in additional increases in the completion of the vaccination, with higher rates of HPV vaccine uptake [6]. Strengthening healthcare providers’ self-efficacy to address the reason for parental hesitancy and routinely recommending the vaccine would be very important ways of improving vaccination rates [11].

Understanding the intention of health professionals to recommend HPV vaccination is critical to establishing an effective immunization plan at the national level. General awareness of HPV prevention and detailed information about the HPV vaccine, including the importance of vaccinating boys and men, should be provided to nurses who are health teachers [27]. Surprisingly, one type of healthcare provider, family pediatricians, was considered a reliable source of information by 83.3% of hesitant parents, and the main factors in hesitancy were not receiving a recommendation to fully vaccinate their child and receiving discordant opinions on vaccinations [13]. For women aged 19–26 who had insurance coverage and access to the HPV vaccine, it was critical for healthcare providers to recommend the vaccine strongly [28]. Appropriate communication between healthcare providers and parents who are hesitant to immunize their child against HPV could change their attitude toward the HPV vaccine [13].

A recent nationwide survey in Korea found that willingness to be vaccinated against HPV decreased from 55.0% in 2007 to 25.8% in 2016 and only 12.1% of men and 22.0% of women knew about the free national HPV vaccination program for girls, launched in June 2016 [29]. Study results showed that unwillingness to get HPV vaccination and lack of awareness of the free HPV vaccine program may have affected the rates of HPV vaccinations, even though a national HPV vaccination initiative was developed [29]. In this atmosphere, education for healthcare providers, especially registered nurses (RNs) who widely serve in public health centers, schools, hospitals, and the community, could be crucial in preventing cervical cancer and encouraging HPV vaccinations. Hence, this study aimed to investigate current perceptions of Korean nurses on HPV vaccination and their intent to vaccinate preteens at the most effective ages.

## Materials and methods

### Study design

We conducted a cross-sectional descriptive study. The survey was performed using a self-administered questionnaire and the data accrued between September 1, 2015 and January 20, 2016.

### Research participants

We recruited nurses to participate in this study using convenience sampling. Three types of nurses—health teachers (who took a teacher-certification test and passed it after being an RN in Korea), public health nurses (who work in a community-based public health center with an RN license), and clinical nurses (who care for patients in a hospital or other healthcare institution with an RN license)—were assumed to take an active role in the prevention of cervical cancer, including HPV vaccination in Korea. At least 50 samples were needed for 10 items (sample: item = 5:1) to conduct factor analysis, and usually 100 to 200 or more cases were strongly recommended for robust factor analysis [30]. Prior to the survey, the research team determined the need for at least 100 participants in each group for the sample to be representative of those groups in Korea.

To sample the representativeness of the three target populations (health teachers, public health nurses, and clinical nurses), we first distributed 240 questionnaires to health teachers in 10 regions of Korea. In 2014, Korea had 7,539 health teachers: 41.7% of all health teachers were in Seoul and Gyeonggi, with 58.3% in other areas. We distributed questionnaires to health teachers proportionately to the two different regional groups. Second, to recruit a representative sample of public health nurses, our research team contacted all public health centers in Korea (*n* = 243) by phone or direct visit: public health centers agreed to take part in this study. We distributed questionnaires by mail to the chief nurse at each public health center. We asked chief nurses to randomly select one nurse working in the health-promotion department in their public health center to answer the questionnaire. Third, we distributed 190 questionnaires to clinical nurses in 20 of the 43 tertiary hospitals in Korea. The target population for clinical nurses was confined to those working in tertiary hospitals in Korea, because they have more resources and have a clinical leadership role as educators in their hospitals. We sampled based on regional proportions of the population; 56% of clinical nurses in Seoul and Gyeonggi, and 44% in other regions. We allocated questionnaires proportionately to hospitals in the two districts. In summary, we distributed a total of 611 questionnaires. We excluded some cases from data analysis, due to incomplete answers.

### Data collection

#### HPV Vaccine Hesitancy

Before commencing the survey, we obtained permission from the original author of the Vaccine-Hesitancy Scale [2]. The Vaccine-Hesitancy Scale consists of 10 items on a 5-point Likert-type scale ranging from 1 to 5. Higher scores indicate positive acceptance of HPV vaccination with a scale of 1 indicating strongly disagree to 5, strongly agree. The tool has the following items: L1. Childhood vaccines are important for my child’s health; L2. Childhood vaccines are effective; L3. Having my child vaccinated is important for the health of others in my community; L4. All childhood vaccines offered by the government programme in my community are beneficial; L5. New vaccines carry more risks than older vaccines; L6. The information I receive about vaccines from the vaccine program is reliable and trustworthy; L7. Getting vaccines is a good way to protect my child/children from disease; L8. Generally I do what my doctor or healthcare provider recommends about vaccines for my child/children.; L9. I am concerned about serious adverse effects of vaccines; L10. My child/children does or do not need vaccines for diseases that are not common anymore.

The original version of the English scale was translated into Korean and back translated to ensure the same meaning by two bilingual experts who have extensive experience with the content of the questionnaire. Finally, we conducted a pretest with 10 nurses, using the translated Korean version to identify grammatical and contextual errors; following the pretest, we modified some sentences to align with cultural values. The Korean scale has the following items: K1. Having adolescent girls vaccinated is important for the health of others in my community; K2. Getting an HPV vaccine is a good way to protect adolescent girls from cervical cancer; K3. Generally, I do what my doctor recommends about vaccines for adolescent girls; K4. The HPV vaccine offered by the government program in my community is beneficial; K5. The HPV vaccine is important for adolescent girls’ health; K6. The HPV vaccine is effective; K7. The information I receive about the HPV vaccine from the vaccine program is reliable and trustworthy; K8. The HPV vaccine carries more risks than other vaccines; K9. I am concerned about serious adverse effects of the HPV vaccine; K10. Adolescent girls do not need the HPV vaccine for cervical cancer, which is no longer common.

Our questionnaire included various sets of the following items. First, we collected the general characteristics of respondents in age, religion, marital status, HPV vaccinated status, education, working type, and work experience. Second, we included opinions about the promotion of HPV vaccination, evaluating perceived importance and perceived confidence and the importance of the nursing role related to HPV vaccination. For this process, we used a 5-point Likert-type scale (1 = not at all to 5 = very much) on the questionnaire. We also inquired about the importance of the nursing role related to HPV vaccination with 4 items: 1) Giving accurate information to students and parents about vaccine efficacy and side effects, 2) Correcting prejudices and wrong ideas about vaccines with students and parents, 3) Assessing vaccine-related knowledge and the positive attitude of nurses, and 4) Making active recommendations to students and parents. Third, we included the Korean version of the HPV Vaccine Hesitancy Scale, consisting of 10 items modified for this study, on the questionnaire. Fourth, we questioned whether respondents agreed to the inclusion of boys, and girls with three types of answers: agreement, disagreement, or neutral. We asked nurses what they thought was the proper age to vaccinate girls and boys with five choices: 9–10, 11–12, 13–15, 16–18, and 19+.

For the health teachers’ survey, one health teachers and four nurses volunteered to distribute and retrieve the questionnaires; for public health nurses, two nurses sent and retrieved questionnaires; and 12 nurses worked to manage and retrieve the questionnaires from clinical nurses. Prior to answering the survey questions, all participants signed an informed-consent form, agreeing to participate in this study. For their participation, we provided a souvenir costing USD 1 when they completed the survey.

### Statistical analysis

We performed factor analysis to test the construct validity of vaccine-hesitancy using the principal axis with the varimax-rotation technique. We calculated eigenvalues and factor loadings for each factor and item. We examined the scale for reliability and internal consistency using Cronbach’s alpha. We analyzed all variables for the descriptive statistics including numbers, percentages, means, and standard deviations. We examined the variables associated with vaccine-hesitancy using the Kruskal–Wallis test and Spearman’s rho coefficients, due to a lack of normalization. We analyzed all data using SPSS version 23.0. Significant levels were considered at *p* < .05.

### Study approval

Seoul National University Institutional Review Board approved this study (IRB No. 1508/002-008).

## Results

### General characteristics of respondents

We distributed a total of 611 questionnaires for this study: 520 were returned (a retrieval rate of 85.1%); six were excluded for analysis because of missing values. Of 514 total respondents, clinical nurses comprised 165 (32.1%), public health nurses were 168 (32.7%), and health teachers were 181 (35.2%) of those answering the survey (see Table 1). Mean age of respondents was 39.32, 58.9% had a religion, and 61.8% were married. The majority (91.2%) of respondents had a bachelor’s or master’s degree in education, and mean working duration was 13.96 years. Of total respondents, 38.5% were vaccinated for HPV.

**Table 1:** Sociodemographic characteristics of participants (*N* = 514).

Characteristics	Category	*n*	%	Mean ± *SD*
Age (year; min-max: 21–62)	21–29	126	24.5	39.32 ± 10.95
	30–39	149	29.0	
	40–49	110	21.4	
	50–62	129	25.1	
Religion	No	211	41.1	
	Yes	303	58.9	
Marital status	Single	196	38.2	
	Married	317	61.8	
HPV vaccine	Not yet vaccinated	315	61.5	
	Vaccinated	197	38.5	
Education	Associate degree	45	8.8	
	Bachelor’s degree	365	71.6	
	Master’s degree	100	19.6	
Working type	Clinical nurse	165	32.1	
	Public health nurse	168	32.7	
	Health teacher	181	35.2	
Working duration (year; min-max: 0.1–38)	<1	21	4.2	13.96 ± 10.90
	1–5	139	27.1	
	6–10	86	16.8	
	11–15	62	12.1	
	16–20	62	12.1	
	21–25	38	7.4	
	26–30	58	11.3	
	>31	47	9.2	

*Note*. Missing values excluded.

In promotion of HPV vaccination, the mean perceived importance was scored at 4.08, and the mean perceived confidence was 3.46 on a 5-point Likert-type scale (see Table 2). Of five items on the importance of the nursing role related to HPV vaccination, giving accurate information to students and parents about vaccine efficacy and side effects acquired the highest mean score of 4.18, followed by correcting prejudices and wrong ideas about vaccines to students and parents, with a mean of 4.15, and vaccine-related knowledge and positive attitude of nurses, with the mean of 4.13. Active recommendations to students and parents were scored with a mean of 3.99; the lowest among five items was the importance of the nursing role to HPV vaccination (see Table 2).

**Table 2:** Opinions about promotion of HPV vaccination, and importance of the nursing role related to HPV vaccination of participants (*N* = 514).

Characteristics	Category	*n*	%	Mean ± *SD*
***Promotion of HPV vaccination***	Perceived importance			4.08 ± 0.65
	Not at all (1)	0	0	
	Not important (2)	4	0.8	
	Do not know (3)	79	15.4	
	Important (4)	303	58.9	
	Very much (5)	128	24.9	
	Perceived confidence			3.46 ± 0.78
	Not at all (1)	3	0.6	
	Not important (2)	54	10.5	
	Do not know (3)	191	37.2	
	Important (4)	238	46.3	
	Very much (5)	28	5.4	
***Importance of nursing role related to HPV vaccination***	Giving accurate information to students and parents about vaccine efficacy and side effects			4.18 ± 0.51
	Not at all (1)	0	0	
	Not important (2)	0	0	
	Do not know (3)	29	5.7	
	Important (4)	361	70.2	
	Very much (5)	124	24.1	
	Correcting the prejudices and wrong ideas about vaccines to students and parents			4.15 ± 0.58
	Not at all (1)	0	0	
	Not important (2)	3	0.6	
	Do not know (3)	46	8.9	
	Important (4)	337	65.6	
	Very much (5)	128	24.9	
	Vaccine-related knowledge and positive attitude of nurses			4.13 ± 0.62
	Not at all (1)	0	0	
	Not important (2)	3	0.6	
	Do not know (3)	59	11.5	
	Important (4)	318	62.0	
	Very much (5)	133	25.9	
	Active recommendation to students and parents			3.99 ± 0.64
	Not at all (1)	1	0.2	
	Not important (2)	6	1.2	
	Do not know (3)	86	16.7	
	Important (4)	327	63.6	
	Very much (5)	94	18.3	

### Validity and reliability of the Vaccine-Hesitancy Scale

For the results of factor analysis, we confirmed adequacy as significant with a Kaiser–Meyer–Olkin value of 0.0877, and a Bartlett’s test of sphericity. The chi-square value was 2861.00 (*p* = .000). The results showed two factors for HPV vaccine-acceptance: positive acceptance consisted of seven items, and negative acceptance consisted of three items. Together, these factors accounting for 67.46% of the variance: individually 47.35%, and 20.11%, respectively (see Table 3).

**Table 3:** Vaccine-acceptance: Factor-analysis and correlations with nurses’ perceptions.

Items	Factor loading		Commonality	*M* ± *SD*	Promotion of HPV vaccination			
					Importance		Confidence	
	Factor 1	Factor 2			*Spearman’s ρ*	*p value*	*Spearman’s ρ*	*p value*
Having adolescent girls vaccinated is important for the health of others in my community	.848		.720	4.02 ± 0.69	0.36	.000	0.35	.000
Getting HPV vaccine is a good way to protect adolescent girls from the cervical cancer	.836		.704	3.99 ± 0.72	0.44	.000	0.41	.000
Generally I do what doctor recommends about vaccine for adolescent girls	.829		.692	3.95 ± 0.75	0.42	.000	0.37	.000
HPV vaccine offered by the government program in my community is beneficial	.822		.683	4.14 ± 0.66	0.40	.000	0.32	.000
HPV vaccine is important for adolescent girls’ health	.819		.686	4.09 ± 0.71	0.35	.000	0.32	.000
HPV vaccine is effective	.811		.660	3.91 ± 0.71	0.32	.000	0.40	.000
The information I receive about HPV vaccine from the vaccine program is reliable and trustworthy	.771		.597	3.58 ± 0.78	0.34	.000	0.45	.000
HPV vaccine carry more risks than other vaccines*		.835	.701	3.05 ± 0.86	0.01	.773	-0.02	.730
I am concerned about serious adverse effects of HPV vaccine*		.807	.654	3.16 ± 0.94	0.07	.115	-0.08	.067
Adolescent girls do not need HPV vaccine for cervical cancer that is not common anymore*		.793	.650	2.40 ± 0.96	-0.08	.060	-0.08	.080
Total (range: 14–49)				37.06 ± 4.94	0.36	.000	0.37	.000
Eigenvalue	4.735	2.011						
Cumulative variance: 67.46%	47.35	20.11						
Cronbach’s α of 10 items: .76	.92	.74						

*Reverse coded in the calculation of the total score.

Among the items measuring HPV vaccine-acceptance, all seven items of positive acceptance significantly correlated with nurses’ perceptions about the importance and their confidence in HPV vaccine promotion. Cronbach’s alpha was .76 for total items; .92 and .74 for subscales of positive acceptance and negative acceptance, respectively (see Table 3).

### Correlations between vaccine-acceptance and perceptions of nurses

Table 3 shows Spearman’s rho correlations among each item for vaccine-acceptance and perceptions, including perceived importance of promoting HPV vaccinations and perceived confidence in promoting HPV vaccination. The total mean score of vaccine-acceptance correlated with perceived importance and perceived confidence, with Spearman’s rho scores of .36, and .37, respectively; both *p*-values were significant at the .05 level. Seven items of positive acceptance were all significant with perceptions of perceived importance and perceived confidence in promoting HPV vaccination. The range of Spearman’s rho results was .32 to .44. “Getting an HPV vaccine is a good way to protect adolescent girls from cervical cancer” presented the highest correlation with perceived importance with a Spearman’s rho of .44, and “The HPV vaccine is effective” showed the lowest correlation with perceived importance, with a Spearman’s rho of .32. In contrast, three items of negative acceptance were all nonsignificant with perceived importance and perceived confidence in promoting HPV vaccination.

### Differences and correlations in nurses’ opinions and demographics

Table 4 shows differences between nurses’ opinions about including girls/boys and best ages, and vaccine-acceptance with *χ*^2^ test statistics calculated from the Kruskal–Wallis test. Nurses positively accepting HPV vaccine (higher mean score in vaccine-acceptance) were more likely to agree with including girls (*χ*^2^ = 91.86, *p* = .000) and including boys for HPV vaccination (*χ*^2^ = 20.13, *p* = .000). Nurses agreed that vaccinating girls and boys showed higher vaccine-acceptance than did nurses who disagreed or were neutral. These results indicate that the Vaccine-Hesitancy Scale in this study met criterion validity. For proper vaccination age, a significant difference emerged between answers for girls and vaccine-acceptance scores among nurses (*χ*^2^ = 74.0, *p* = .000), whereas no difference arose between answers on the proper vaccination age of boys and vaccine-acceptance scores (*χ*^2^ = 8.43, *p* = .077).

**Table 4:** Vaccine-acceptance: Differences by nurses’ opinion (*N* = 514).

Characteristic	Category	*n*	%	Vaccine-acceptance *M ± SD*	*χ*^2^*	*p value*
*Vaccinated subjects*						
Including girls	Agreement	427	83.6	38.03 ± 4.18	91.96	.000
	Disagreement	15	2.9	27.93±7.10		
	Neutrality	69	13.5	33.04 ± 4.71		
Including boys	Agreement	278	54.4	38.05 ± 4.10	20.13	.000
	Disagreement	53	10.4	34.51 ± 6.72		
	Neutrality	180	35.2	36.29 ± 5.12		
*Proper vaccination age*						
Girls (yr)	9–10	40	7.9	36.83 ± 5.12	74.00	.000
	11–12	121	24.0	38.93 ± 4.38		
	13–15	161	31.9	38.27 ± 4.34		
	16–18	122	24.1	36.50 ± 3.63		
	≥ 19	61	12.1	32.03 ± 5.78		
Boys (yr)	9–10	27	5.4	36.96 ± 6.03	8.43	.077
	11–12	81	16.2	38.64 ± 4.49		
	13–15	123	24.6	37.41 ± 4.25		
	16–18	150	30.0	36.83 ± 3.62		
	≥ 19	119	23.8	36.27 ± 6.61		

*Note*. Missing-values were excluded.

* calculated from the Kruskal–Wallis test in the SPSS package.

Table 5 shows Spearman’s rho correlation results between vaccine-acceptance and demographic factors. Nurse respondents’ vaccinated status positively related to intentions toward HPV vaccinations (Spearman’s rho = .19, *p* = .000), although correlations with age, religion, marital status, education, and working duration were not significant.

**Table 5:** Vaccine-acceptance: Correlations by nurses’ demographics (*N* = 514).

Category	Spearman’s rho	*p value*
Age	0.03	.515
Religion*	0.06	.173
Marital status*	0.00	.961
HPV vaccine*	0.19	.000
Education	0.06	.151
Working duration	0.04	.324

*Note*. Missing-values were excluded.

* Dummy variables.

## Discussion

This was the first study to unveil nurses’ perceptions of HPV vaccination and intentions toward HPV vaccination among Korean nurses, using the Vaccine-Hesitancy Scale developed by Larson et al. [2]. The strength of this study was in recruiting three types of nurses—clinical nurses, public health nurses, and health teachers—who are caring for adolescent populations in the Republic of Korea. To design clinical or community-based interventions to increase the overall rates of HPV vaccinations in Korea, the most critical evidence was understanding actual healthcare providers’ attitudes; especially their hesitancy toward HPV vaccinations.

Although the Republic of Korea recently started a free HPV vaccination program with a small target group (all 12-year old girls born between January 1, 2003 and December 31, 2004; total 464,932) in June 20, 2016, only 27.8% were vaccinated with the new free HPV vaccine as of November 2016 [18]. To assure the effectiveness of the nationally initiated immunization program and the better protection of adolescents against HPV-associated infections, the professional commitment to increase the knowledge of HPV infection and vaccination among public health nurses, health teachers, and general practitioners who are responsible for adolescents’ primary care in HPV-attributable cancer prevention needed to proceed [6,16,31]. Further, providing proper and timely support to healthcare professionals determines the success of implementing the program; it is critical to encourage healthcare providers to participate in the programe with timely information about the disease, the vaccine, and the program, to increase consensus and create a more positive culture in acceptance of HPV vaccination [32]. Many countries have shown significant differences in the sustainability of national immunization programs because a well-planned vaccination program needs to consider local cultural perceptions and sensitivities [32]. To implement national immunization programs successfully, researchers highly recommend developing feasible action plans regarding implementation [32].

Several studies showed that a strong recommendation from physicians to receive the HPV vaccine as a safe, routine immunization prevents cervical cancers and other sexually transmitted diseases and enhances vaccine uptake [20, 27]. A French study also showed that although healthcare providers frequently recommended the HPV vaccine, general practitioners were still hesitant, due to unfavorable perceptions of its risk–benefit balance, a decision not to vaccinate one’s own daughter with this vaccine, and doubt about vaccine utility in general [25]. Although the acceptance of vaccination is an outcome behavior resulting from a complex decision-making process that a wide range of factors can potentially influence [3], several studies reported that one target intervention was effective for HPV vaccine uptake: a strong recommendation from the healthcare provider; particularly the physician [28,33]. Healthcare providers, including physicians, nurses, and health teachers who are caring for adolescents’ health should take an active role in educating adolescents and their parents, providing clear information, and showing firm attitudes in recommending HPV vaccinations.

Study results revealed that nurses who are more hesitant about the HPV vaccine have disagreed with the inclusion of boys in the HPV vaccination program. Although many countries are integrating HPV vaccinations into their national health program [17], only two countries—Australia [34] and the United States [35]—officially provide the vaccine to boys and girls; all other countries established HPV vaccination programs only for girls. In a longitudinal national study conducted in the United States, only 2% of sons aged 11 to 17 had received any doses of HPV vaccine at baseline; researchers concluded that physician recommendations and education about HPV vaccine for boys are key strategies for improving vaccination rates [36]. A statewide study also disclosed that far fewer (46%) healthcare providers routinely recommend HPV vaccine for boys than for girls [11]. A Canadian study yielded inconclusive results in including boys for HPV vaccine uptake as universal coverage [33]. Although our finding supports that reasons for vaccinating boys are much less distinct, more disagreement was found among Korean nurses; many studies documented that vaccination of both sexes is strategically important to prevent HPV-attributable diseases for children and their future partners [15, 37, 38].

A study of school nurses’ HPV vaccine attitudes for adolescents and their parents showed that higher levels of knowledge on HPV and its vaccine predicts more positive attitudes toward the HPV vaccine; however, the strongest predictor was the perception of role as an opinion leader [16]. The study raised the need for professional development for school nurses to increase HPV vaccine knowledge and to encourage a positive attitude to engage with parents and adolescents to recommend HPV vaccination [16]. Another study reported that the HPV vaccination rate of respondents was 38.5%, whereas other studies reported a lower HPV vaccination rate in Korea, with only 5% of women vaccinated among university students [39]. HPV awareness was also low among men and women [40], and HPV knowledge was even found to be insufficient among clinical nurses [41] and health teachers [27] in Korea. Only 23% of Korean health teachers who worked in elementary, middle, high, and special schools in Korea (*n* = 757) answered they have ever taught about HPV [27]. All nurses need more education about HPV and HPV vaccination [8]; researchers found that 56% of nurses reported they needed more education about HPV [42].

Knowledge had a positive effect on intention and self-efficacy of school nurses, and attitude had a positive impact on perceptions of their role as opinion leaders [43]. Interventions to increase HPV vaccination should focus on increasing knowledge, attitude, and intention, by designing education programs guiding parents and children about the benefits of HPV vaccination and its safety [43]. Researchers found that a higher level of knowledge helped predict school nurses’ positive attitudes, but was not the strongest predictor [34]. Rather perceived role as an opinion leader for HPV vaccine strongly predicted school nurses’ positive attitude [16].

Although a Canadian study suggested nurse practitioners as primary care providers collaborate with public health agencies to expand knowledge and coverage related to HPV vaccine [33], our study suggests RNs take charge of recommending HPV vaccines and increase their vaccine-related knowledge to protect adolescents against HPV-related diseases. Swedish researchers noted that parents had contacted 90% of school nurses with questions about the vaccine and most questions related to vaccine safety [42]. It is necessary to continuously provide professional education and training for nurses who could be key people delivering coherent information regarding HPV vaccination to parents and adolescents [13, 42].

This study has some limitations to consider. First, this study was conducted before the national HPV vaccination program started, with a time lag of 5 to 9 months. Nurses may have experienced some perception changes after the vaccination program was initiated. In the future, a study considering the effect of adopting the national program could yield a clearer picture of how to develop effective interventions for HPV vaccinations. Second, although the scale was developed to investigate the level of *vaccine-hesitancy*, higher scores on the scale indicated greater willingness to accept the vaccine; thus, our study used the term *vaccine-acceptance* for tables, explanations, and interpretations to clarify meanings. Third, we performed convenience sampling to determine the number of cases according to the type of nurses and their regional distributions. A more systematic method of sampling based on geographical stratification would better represent samples and minimize selection bias.

## Conclusions

In this study, we developed a Korean version of the Vaccine-Hesitancy Scale related to the HPV vaccine, validated by nurses in Korea. This study showed that vaccine-acceptance levels of HPV vaccination among nurses significantly align with nurses’ attitudes and opinions about HPV vaccination for girls and boys.

Countries, including the Republic of Korea, have established national HPV vaccination programs as a strategy to encourage larger populations—female and male—to be vaccinated. However, most countries only covered 11- to 12-year-old girls’ vaccinations. Including adolescent boys is also very important to protect all children from possible sexual transmission of HPV. This study provides clear evidence that healthcare providers’ attitudes and confidence affect vaccine acceptance. Further research is needed to investigate changing patterns of healthcare providers’ attitudes and vaccine-uptake status for a larger range of adolescent populations, following the adoption of the national program. The goal is to design clinical and community-based interventions for HPV vaccination at the national level. As educators and healthcare providers caring for adolescent populations, nurses should take an active role in promoting HPV vaccination and teaching prevention strategies against HPV infections. Reducing HPV vaccine-hesitancy requires continuing-education programs to strengthen the educational role of healthcare providers on HPV vaccination. Such educational programs would support primary prevention of cervical cancer and other related diseases. Additionally, frequently providing updated information about the HPV vaccine to healthcare providers and health teachers will help them develop a strong positive attitude about HPV vaccination. Empowering, educating, and motivating nurses on the need for primary prevention against HPV infections with HPV vaccinations will help promote people accepting the HPV vaccine and will motivate nurses to communicate with parents more actively. More future research is needed to develop advanced interventions to promote HPV vaccination in various clinical settings.

## Supporting information

S1 Data Koreannurses514_1. (SAV)
